# Effect of zinc-containing pessaries on menopausal genitourinary syndrome, a double-blind randomised trial

**DOI:** 10.1186/s12905-025-04037-y

**Published:** 2025-10-03

**Authors:** Assem Mousa, Ismail Elgarhi, Bahaa Ibrahim, Emad Atalla, Mazen Abdel-Rasheed, Omar Nagy, Kareem Shaheen

**Affiliations:** 1 Obstetrics and Gynaecology Department, Faculty of Medicine, Al-Azhar University, Cairo, Egypt; 2 Obstetrics and Gynaecology Department, Ashmoun Central Hospital, Elmenofia, Egypt; 3 Reproductive Health Research Department, National Research Centre, 33 El-Buhouth St, Dokki, Cairo, 12622 Egypt; 4 Obstetrics and Gynaecology Department, Faculty of Medicine, Minia University, Minia, Egypt

**Keywords:** Genitourinary syndrome of menopause, Vulvovaginal atrophy, Atrophic vaginitis, Zinc pessaries

## Abstract

**Background:**

Menopause-related changes in the vulva, vagina, and lower urinary tract are called the genitourinary syndrome of menopause (GSM). Symptoms include urgency, dysuria, recurrent urinary tract infection, dryness, burning, irritation in the genitals, and sexual symptoms. The purpose of this study is to evaluate the efficacy of zinc-containing vaginal pessaries in relieving GSM symptoms and to document any adverse reactions that may occur.

**Methods:**

A pilot prospective randomised study was conducted on fifty postmenopausal women. Group 1 of 25 cases received zinc-containing pessaries, and Group 2 of 25 cases received placebo pessaries. Both groups received the treatment regimen in the form of one pessary per vagina before bedtime for 14 consecutive nights. GSM symptoms on a visual analog scale (VAS) and Vaginal Health Index (VHI) were reported before and after treatment.

**Result:**

Regarding VHI values after treatment, patients treated with zinc pessaries showed significant improvement regarding elasticity, fluid secretion, vaginal pH, epithelial mucosa, moisture, and the total score compared to those treated with placebo pessaries. Regarding the VAS score, also patients treated with zinc pessaries had statistically significant improvement regarding vaginal pain, burning sensation, itching, vaginal dryness, dyspareunia, dysuria, and the total score compared to those treated with placebo pessaries.

**Conclusion:**

patients of GSM treated with zinc pessaries had higher satisfaction with significant improvement in the VHI and VAS scores.

## Introduction

Vulvovaginal atrophy (VVA) is characterised by dryness, burning, itching, discomfort, and sometimes dyspareunia, affecting up to 40% of postmenopausal women [1, 2]. However, terms like “vulvovaginal atrophy” and “atrophic vaginitis” are limited as they focus solely on genital symptoms, omitting urinary symptoms, and incorrectly imply inflammation or infection.

Therefore, the term “genitourinary syndrome of menopause” (GSM) has replaced these medical terms as recommended by the “International Society for the Study of Women’s Sexual Health” (ISSWSH) and the “North American Menopause Society” (NAMS) in 2014 [3]. GSM symptoms include genital symptoms (dryness, burning, and irritation), urinary symptoms (urgency, dysuria, and urinary tract infections), and sexual symptoms (lack of lubrication, discomfort, and dysfunction) [4].

Reduced oestrogen levels due to ovarian ageing contribute to GSM pathophysiology. Vaginal oestrogen supplementation has long been the gold standard for treating these menopausal symptoms. However, many women are unwilling to or uncomfortable with this approach. Selective oestrogen receptor modulators (SERMs) also offer therapeutic benefits by reducing urinary urgency and enhancing patient quality of life [5]. Vaginal dryness, one of the most unpleasant signs of VVA, can be managed with various non-hormonal therapy techniques. According to the North American Menopause Society’s 2013 policy statement, non-hormonal vaginal lubricants and moisturisers and regular sexual activity should treat VVA symptoms first [6].

Zinc is recognised to be the primary constituent of a large number of metalloenzymes. It also plays an essential part in healing, the biosynthesis process, and the homeostasis of different connective tissues [7]. Zinc has a beneficial influence on producing extracellular components in human vaginal smooth muscle cells. Previous research investigated the role of vaginal topical Zinc on vaginal and vulvar remodelling, with an increase in the amount of elastin created by human vaginal smooth muscle cells. Additionally, past studies conducted on animals have demonstrated that Zinc is an essential component in forming the vaginal extracellular matrix [7–9].

In this study, we aimed to assess the beneficial influence of zinc-containing pessaries on GSM/VVA symptoms and to document any adverse reactions that may occur.

## Methods

A pilot study was conducted from December 2021 to December 2022‎ at Ashmoun Central Hospital in Elmenofia on fifty postmenopausal women under the supervision of the obstetrics and gynaecology staff of the Faculty of Medicine, Al Azhar University. Registration at ClinicalTrials.gov (NCT05453227) was completed on 12/07/2022. Clear consent was obtained from every patient after the aim of the research was explained.

Inclusion criteria included postmenopausal women with signs of vulvovaginal atrophy or GSM, such as vaginal dryness, burning, itching, vaginal pain, and dyspareunia. Postmenopause was defined as amenorrhea for at least 12 months without a clear cause or a persistently increased serum follicle-stimulating hormone level of more than 30 mIU/mL.

Exclusion criteria involved those patients who have a history of breast or gynaecological cancer, those patients with pelvic organ prolapse, those patients who have used any vaginal product or douching within the past three months, and those patients who have used any form of local or systemic hormone treatment within the past 6 months.

The fifty postmenopausal women were randomly distributed into 2 groups using computer-generated random numbers. Group 1 included 25 cases who received medicated pessaries with the active ingredient (zinc sulphate), and Group 2 included 25 cases who received treatment with placebo pessaries. Both groups received the treatment regimen in the form of one pessary per vagina before bedtime for 14 consecutive nights.

Identical-appearing zinc sulphate and placebo pessaries were manufactured using the same glycerol-gelatine base. Computer-generated randomisation codes were held by an independent pharmacy unit at Cairo University. Treatment assignments were concealed in sequentially numbered, opaque containers. Participants, clinicians, and outcome assessors were therefore blinded to group allocation. Unblinding occurred after completion of all assessments for the final analysis.

Pessaries were manufactured at the Drug Manufacturing Unit at the Faculty of Pharmacy, Cairo University, Egypt. Two sets of pessaries were prepared. Placebo pessaries (plain glycerol-gelatine base) were prepared according to the US Pharmacopoeia and consisted of 20, 70 and 10% of gelatine, glycerol and distilled water, respectively. Medicated pessaries were prepared the same way by dissolving 1 part of zinc sulphate and two parts of lactic acid in the aqueous portion of 97% of the base. Pessaries were kept in tight containers at ambient temperature (below 35 °C).

Postmenopausal women with GSM symptoms were asked to report their symptoms (vaginal pain, burning sensation, itching, vaginal dryness, dyspareunia, and dysuria) and mark the severity of these symptoms on a 0–10 visual analog scale (VAS). A score of 0 indicated an absent symptom, and a score of 10 indicated the worst possible symptom. This assessment was done before starting treatment and after completion of treatment.

Clinical data regarding the Vaginal Health Index (VHI) score components were gathered, including vaginal elasticity, fluid secretion, pH and epithelial mucosa and vaginal hydration. Each component was scored on a scale of 1 (worst) to 5 (best); so lower scores indicated more severe atrophy (Bachmann et al. 1994). The Vaginal Maturation Index (VMI) was evaluated by calculating the percentage of superficial, intermediate and parabasal epithelial cells on the smear. So, we calculated the VMI according to the following formula [(1× %superficial) + (0.5 × %intermediate) + (0 × % parabasal)]. Both VHI and VMI were also calculated before starting treatment and after completion of treatment.

All participants completed a satisfaction questionnaire two weeks after completion of the study to give their feedback. These questions were:

- “Q1: Have you experienced any side effects during or after the completion of the treatment?” (Yes/No).
- “Q2: How satisfied were you with the moisturising effect of the topical?” (depending on vaginal health score on a scale of 1 to 5).
- “Q3: How many days did the moisturising effect last after stopping the treatment?”
- “Q4: Did your vulvovaginal symptoms improve significantly?” (Yes/No).
- “Q5: Did you feel more self-confident after using the topical?” (Yes/No).
- “Q6: Are you satisfied with the consistency of the topical?” (Yes/No).

The primary outcome was to assess the effect of a novel zinc-containing vaginal topical on GSM/VVA symptoms.

### Statistical analysis

Data was collected, tabulated, and analysed with the use of SPSS 22.0 for Windows (SPSS Inc., Chicago, Illinois, USA). The Chi-square test (χ2) and the Fisher exact test were utilised to ascertain the existence of differences between qualitative variables. Means ± standard deviation (SD), medians and ranges were utilised for the analysis of quantitative variables. The independent T-test was utilised to make comparisons between the two groups for quantitative variables. P-value was used to assess the significant differences; if less than 0.05, then the difference was considered statistically significant.

## Results

**Fig. 1:**
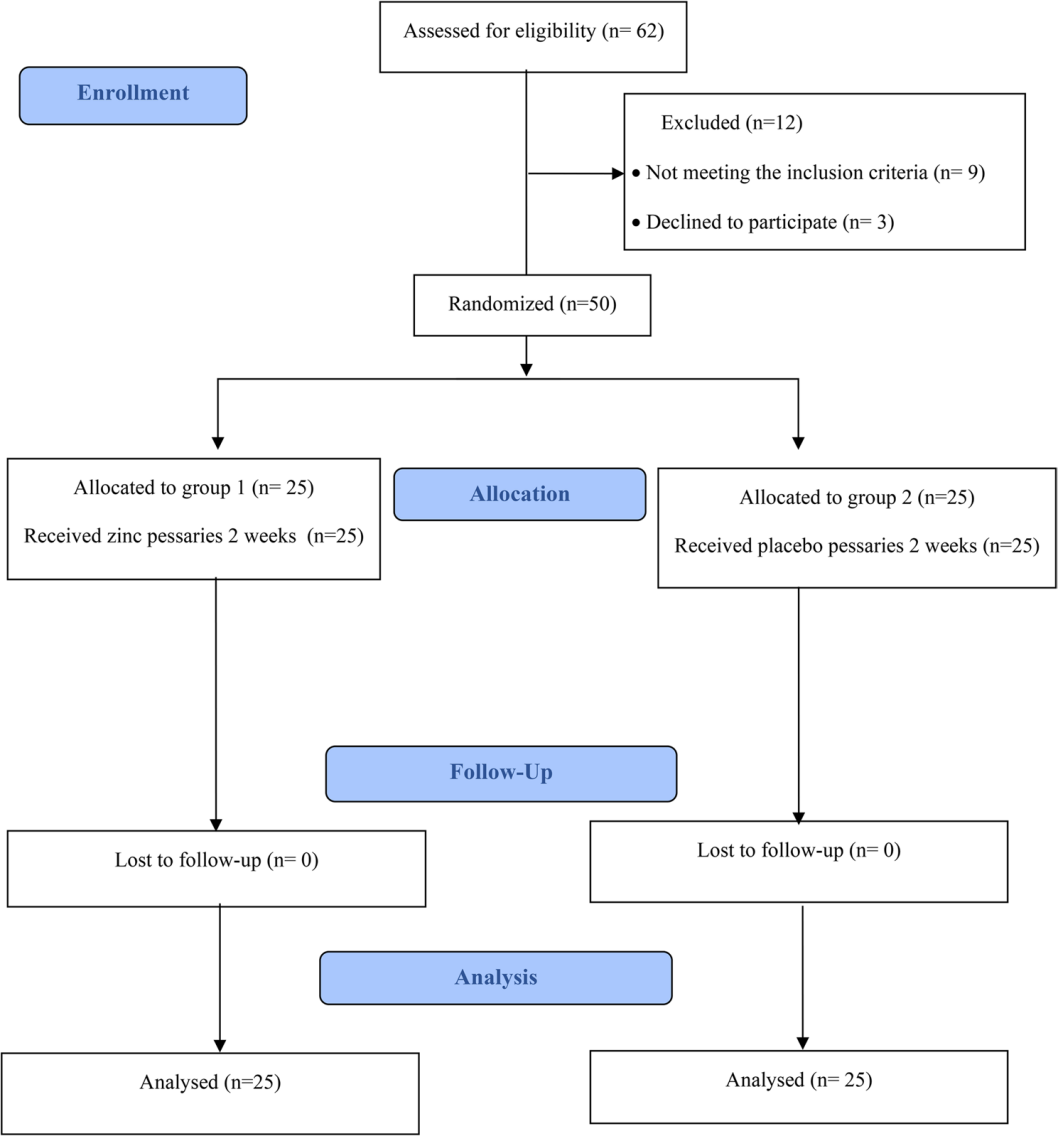
Flowchart of participants in the study

Fifty postmenopausal women were enrolled in this clinical trial following the CONSORT guidelines, as shown in Fig. 1. There was no statistically significant difference in age, BMI, parity, menopausal duration, or residence among the groups, as shown in Table 1.

**Table 1:** Comparison of the groups’ demographics

		Group 1 (*n* = 25)	Group 2 (*n* = 25)	*P*- value
Age	Mean ± SD	59.92 ± 2.45	60.16 ± 2.82	0.750
Years after menopause	Mean ± SD	10.80 ± 2.92	10.96 ± 3.06	0.851
BMI	Mean ± SD	30.84 ± 1.98	31.54 ± 2.46	0.273
Parity	Median (Range)	2 (1–4)	3 (0–4)	0.172
Residence	Rural Urban	15 (60%) 10 (40%)	13 (52%) 12 (48%)	0.569

Both groups were assessed before treatment. The VAS score revealed no statistically significant difference before treatment among the two groups regarding vaginal pain, burning sensation, itching, vaginal dryness, dyspareunia, and dysuria. In addition, the VHI revealed no statistically significant difference before treatment among the two groups regarding elasticity, fluid secretion, vaginal pH, epithelial mucosa, moisture, and the total score. Also, there was no statistically significant difference regarding the VMI, as shown in Table 2.

**Table 2:** Comparison between the two groups before treatment

			Group 1 (*n* = 25)	Group 2 (*n* = 25)	*P*- value
VAS score	Vaginal pain	Mean ± SD Median (Range)	4.48 ± 1.23 4 (3–6)	4.36 ± 1.04 4 (3–6)	0.711
	Burning sensation	Mean ± SD Median (Range)	4.00 ± 1.44 4 (2–6)	3.84 ± 1.43 3 (2–6)	0.696
	Itching	Mean ± SD Median (Range)	5.80 ± 1.73 6 (3–8)	6.00 ± 1.47 6 (3–8)	0.662
	Vaginal dryness	Mean ± SD Median (Range)	7.20 ± 1.91 7 (4–10)	7.60 ± 2.14 8 (4–10)	0.490
	Dyspareunia	Mean ± SD Median (Range)	6.32 ± 1.57 6 (4–9)	5.92 ± 1.80 6 (4–9)	0.407
	Dysuria	Mean ± SD Median (Range)	0.68 ± 0.69 1 (0–2)	1.08 ± 0.76 1 (0–2)	0.057
Vaginal health index	Elasticity	Mean ± SD Median (Range)	1.84 ± 0.80 2 (1–3)	2.00 ± 0.76 2 (1–3)	0.473
	Fluid secretion	Mean ± SD Median (Range)	2.16 ± 0.75 2 (1–3)	2.24 ± 0.83 2 (1–3)	0.722
	Vaginal pH	Mean ± SD Median (Range)	6.12 ± 0.88 6 (5–7)	5.96 ± 0.84 6 (5–7)	0.514
	Epithelial mucosa	Mean ± SD Median (Range)	2.16 ± 0.69 2 (1–3)	1.96 ± 0.79 2 (1–3)	0.344
	Moisture	Mean ± SD Median (Range)	2.08 ± 0.81 2 (1–3)	2.00 ± 0.87 2 (1–3)	0.738
Vaginal Maturation Index		Mean ± SD Median (Range)	45.64 ± 11.61 44 (20.5–72)	51.66 ± 9.51 52 (31.5–68)	0.051

After treatment, both groups were assessed again. Regarding the VAS score, a statistically significant difference was found between the two groups regarding vaginal pain, burning sensation, itching, vaginal dryness, dyspareunia, and dysuria, as shown in Table 3. The VHI revealed statistically significant differences after treatment among the two groups regarding elasticity, fluid secretion, vaginal pH, epithelial mucosa, and moisture. However, there was no statistically significant difference regarding the VMI. The satisfaction questionnaire after treatment revealed that patients in group 1 were significantly more satisfied and improved compared to those in group 2, as shown in Table 4.

**Table 3:** Comparison between the two groups after treatment

			Group 1 (*n* = 25)	Group 2 (*n* = 25)	*P*- value
VAS score	Vaginal pain	Mean ± SD Median (Range)	1.52 ± 1.08 1 (0–3)	3.48 ± 1.29 4 (2–5)	< 0.001
	Burning sensation	Mean ± SD Median (Range)	1.80 ± 1.44 2 (0–4)	3.48 ± 1.45 4 (1–5)	< 0.001
	Itching	Mean ± SD Median (Range)	1.12 ± 0.83 1 (0–2)	2.44 ± 1.23 2 (1–4)	< 0.001
	Vaginal dryness	Mean ± SD Median (Range)	2.12 ± 1.51 2 (0–4)	3.92 ± 1.29 4 (2–6)	< 0.001
	Dyspareunia	Mean ± SD Median (Range)	1.52 ± 1.05 2 (0–3)	4.52 ± 1.45 4 (2–7)	< 0.001
	Dysuria	Mean ± SD Median (Range)	0.48 ± 0.51 0 (0–1)	1.00 ± 0.87 1 (0–2)	0.013
Vaginal health index	Elasticity	Mean ± SD Median (Range)	3.32 ± 1.03 3 (2–5)	2.68 ± 0.48 3 (2–3)	0.007
	Fluid secretion	Mean ± SD Median (Range)	3.72 ± 1.17 4 (2–5)	2.20 ± 0.41 2 (2–3)	< 0.001
	Vaginal pH	Mean ± SD Median (Range)	4.68 ± 0.85 5 (3–6)	5.16 ± 0.75 5 (4–7)	0.039
	Epithelial mucosa	Mean ± SD Median (Range)	3.92 ± 1.12 4 (2–5)	2.64 ± 0.49 3 (2–3)	< 0.001
	Moisture	Mean ± SD Median (Range)	2.96 ± 1.06 3 (2–5)	2.44 ± 0.51 2 (2–3)	0.032
Vaginal Maturation Index		Mean ± SD Median (Range)	47.64 ± 11.72 45.5 (22.5–73.5)	52.52 ± 9.75 53 (31.5–69.5)	0.116

**Table 4:** Clarification of the questionnaire after treatment between the two groups

		Group 1 (*n* = 25)	Group 2 (*n* = 25)	*P*- value
Q1: Have you experienced any side effects during or after completion of the treatment? ^a^	Yes No	6 (24%) 19 (76%)	13 (52%) 12 (48%)	0.041
Q2: How satisfied were you with the moisturising effect of the topical?	Median (Range)	4 (3–5)	2 (1–3)	< 0.001
Q3: How many days did the moisturising effect last after stopping the treatment?	Median (Range)	3 (1–5)	2 (1–4)	0.001
Q4: Did your vulvovaginal symptoms improve significantly?	Yes No	21 (84%) 4 (16%)	4 (16%) 21 (84%)	< 0.001
Q5: Did you feel more self-confident after using the topical?	Yes No	22 (88%) 3 (12%)	4 (16%) 21 (84%)	< 0.001
Q6: Are you satisfied with the consistency of the topical?	Yes No	20 (80%) 5 (20%)	20 (80%) 5 (20%)	1

^a^Side effects included application-site burning, itching, irritation

## Discussion

GSM is now used to describe symptoms of physical changes in the vulva, vagina and lower urinary tract related to menopausal oestrogen deficiency. In this study, we aimed to test the effect of novel zinc-containing vaginal pessaries on GSM symptoms. A survey was done on 3046 US postmenopausal women. This survey indicated that dryness (55%), dyspareunia (44%), and irritation (37%) are the most common GSM symptoms and affect sexual satisfaction in 59% of participants [10].

Several studies have explored non-hormonal approaches to relieve GSM symptoms aimed at mitigating symptoms associated with vaginal atrophy. These non-hormonal interventions encompass a range of lubricants and vaginal moisturisers. Regular application of long-acting vaginal moisturisers has been demonstrated to significantly ameliorate vaginal dryness and reduce vaginal pH to levels observed in premenopausal individuals [11, 12]. However, these effects occur without substantial alteration in the VMI and are comparatively less efficacious than hormonal therapies [13]. In addition, there is a paucity of rigorous clinical trials assessing their safety and therapeutic efficacy [14].

Takacs et al. investigated how vaginal zinc supplementation affected vaginal remodelling. They discovered that at 20 µM zinc tissue level, Zinc has a favourable influence on the creation of extracellular components generated by the muscle, consequently boosting the quantity of elastin produced in human vaginal smooth muscle cells. They suggest that gel containing Zinc play a dual role—both structural and symptomatic—in improving the vaginal microenvironment in postmenopausal women [7, 15]. This aligns with our findings, highlighting the therapeutic potential of Zinc as a non-hormonal intervention for GSM. Our clinical trial extends this evidence by demonstrating that zinc pessaries significantly improved GSM symptoms.

Similarly, previous animal studies have demonstrated that Zinc is a key component of the extracellular matrix present in vaginal tissue. When rats were fed a zinc-deficient diet, their vaginal architecture was altered to mirror menopausal status, and when Zinc was provided vaginally, the vaginal extracellular matrix was reconstructed with qualities equivalent to those of adolescent rats [7, 8]. Furthermore, in humans, zinc tissue levels in the uterus are at their lowest after menopause, and low zinc levels may have a role in the development of atrophic alterations [16].

The zinc sulphate heptahydrate complex exhibits a strong affinity for water molecules, coordinating seven molecules to facilitate adequate hydration of the atrophic vaginal epithelium. Additionally, Zinc has the capacity to permeate the vaginal mucosa, potentially reaching deeper histological layers, including the lamina propria and muscularis, where it may exert localised effects [17].

The patients treated with zinc pessaries in our study were significantly more satisfied and improved than those treated with placebo pessaries. In line with our findings, Takacs et al. (2019) found that 97% of women had well-moisturised vagina during treatment with topical Zinc, and 94% reported that the treatment was quite pleasant. In 75% of women, the moisturising effect remained at least one week after therapy was stopped. After using the gel, 82% of subjects felt more confident and reported a substantial increase in the quality of their sexual connection. The vaginal gel therapy was very or satisfactorily received by 92% of women. The gel’s consistency was acceptable to 85% of the women, and the scent was acceptable to all of them. Side effects were uncommon and modest [15].

In our study, the improvement in VAS scores was evident across all symptoms evaluated, including vaginal pain, burning sensation, itching, vaginal dryness, dyspareunia, and dysuria. In contrast, the findings reported by Takacs et al. (2019) demonstrated statistically significant VAS score improvements for burning sensation, itching, vaginal dryness, and dyspareunia, while the changes observed in vaginal pain and dysuria did not reach statistical significance [15].

According to Hatem et al. (2013), the most common mechanism of Zinc used as an anti-irritant product is that zinc salts can prevent irritation by binding to negatively charged regions exposed on the surface of proteins and altering the charge configuration of the protein, thereby preventing subsequent protein-protein interactions between irritants and exposed mucous membranes [18].

Damjanovich et al. (2020) studied the beneficial effect of topical Zinc in 82 menopausal women. The overall VAS score was 14 ± 14, while the mean VHI was 15 ± 6. They discovered substantial correlations between cervicovaginal lavage zinc levels and age, menopausal state, vaginal dryness, and vaginal atrophy. Correlation analysis also revealed a somewhat positive link between VMI and cervicovaginal lavage zinc levels (*P* < 0.01). Only vaginal atrophy remained significant in the multivariate regression model, which included all relevant covariates [19].

Our study builds upon these findings by confirming the symptomatic benefits of vaginal zinc supplementation using a pessary formulation, and by demonstrating measurable improvement in both subjective (VAS scores) and objective (VHI, VMI) outcomes. The significance of our findings lies in the potential utility of zinc pessaries as a practical, non-hormonal option for managing GSM, especially for women who cannot or prefer not to use estrogen-based therapies. This could expand the range of available treatments in clinical practice. Future studies should explore long-term outcomes, patient adherence, and comparative effectiveness with other non-hormonal interventions.

The main limitation is that this study is a pilot one with only two weeks of treatment and no follow-up period. In addition, our formulation contained 1 part zinc sulphate and 2 parts lactic acid; however, Zinc was the primary active component. On the contrary, the strength of our study is using a different formula of topical Zinc in the form of vaginal pessaries that are theoretically easier to apply than the topical gel. Different subjective and objective outcomes were used to assess Zinc-containing pessaries for GSM symptoms.

## Conclusion

Our study concluded that zinc pessaries provide a non-hormonal therapeutic option for cases of GSM. Zinc pessaries significantly improved patient satisfaction and the VAS scores compared to the placebo group.

## Data Availability

The data that support the findings of this study are available from Ashmoun Central Hospital in Elmenofia, but restrictions apply to the availability of these data, which were used under license for the current study, and so are not publicly available. Data are, however, available from the corresponding author upon reasonable request and with permission of Ashmoun Central Hospital.

## References

[1] Gandhi J, Chen A, Dagur G, Suh Y, Smith N, Cali B, et al. Genitourinary syndrome of menopause: an overview of clinical manifestations, pathophysiology, etiology, evaluation, and management. Am J Obstet Gynecol. 2016;215:704–11. 27472999 10.1016/j.ajog.2016.07.045

[2] Kim H-K, Kang S-Y, Chung Y-J, Kim J-H, Kim M-R. The recent review of the genitourinary syndrome of menopause. J Menopausal Med. 2015;21:65–71. 26357643 10.6118/jmm.2015.21.2.65 PMC4561742

[3] Portman DJ, Gass MLS, Vulvovaginal Atrophy Terminology Consensus Conference Panel. Genitourinary syndrome of menopause: new terminology for vulvovaginal atrophy from the International Society for the Study of Women’s Sexual Health and the North American Menopause Society. Menopause. 2014;21(10):1063–8. 25160739 10.1097/GME.0000000000000329

[4] Nappi RE, Martini E, Cucinella L, Martella S, Tiranini L, Inzoli A, et al. Addressing vulvovaginal atrophy (VVA)/genitourinary syndrome of menopause (GSM) for healthy aging in women. Front Endocrinol. 2019;10:561. 10.3389/fendo.2019.00561 PMC6712495 31496993

[5] Schiavi MC, D’Oria O, Aleksa N, Vena F, Prata G, Di Tucci C, et al. Usefulness of ospemifene in the treatment of urgency in menopausal patients affected by mixed urinary incontinence underwent mid-urethral slings surgery. Gynecol Endocrinol. 2019;35:155–9. 30324854 10.1080/09513590.2018.1500534

[6] North American Menopause Society. Management of symptomatic vulvovaginal atrophy: 2013 position statement of the North American menopause society. Menopause. 2013;20:888–902. 23985562 10.1097/GME.0b013e3182a122c2

[7] Takacs P, Zhang Y, Candiotti K, Jaramillo S, Medina CA. Effects of PPAR-delta agonist and zinc on vaginal smooth muscle cells collagen and tropoelastin production. Int Urogynecol J. 2012;23:1775–9. 22576330 10.1007/s00192-012-1807-y

[8] Takacs P, Jaramillo S, Zhang Y, Datar R, Williams A, Olczyk J, et al. The effects of PPARδ agonist and zinc on ovariectomised rats’ vagina. Urogynecology. 2013;19:126–31. 10.1097/SPV.0b013e31828746e9 23611928

[9] Csikós A, Kozma B, Pór Á, Kovács I, Lampé R, Miklós I, et al. Zinc transporter 9 (SLC30A9) expression is decreased in the vaginal tissues of menopausal women. Biol Trace Elem Res. 2021;199:4011–9. 33409913 10.1007/s12011-020-02525-w

[10] Wysocki S, Kingsberg S, Krychman M. Management of vaginal atrophy: implications from the REVIVE survey. Clin Med Insights Reprod Health. 2014;8:23–30. 24987271 10.4137/CMRH.S14498 PMC4071759

[11] Lee Y-K, Chung HH, Kim JW, Park N-H, Song Y-S, Kang S-B. Vaginal pH-balanced gel for the control of atrophic vaginitis among breast cancer survivors: a randomised controlled trial. Obstet Gynecol. 2011;117:922–7. 21422866 10.1097/AOG.0b013e3182118790

[12] Kim SH, Park ES, Kim TH. Rejuvenation using platelet-rich plasma and lipofilling for vaginal atrophy and lichen sclerosus. J Menopausal Med. 2017;23:63–8. 28523261 10.6118/jmm.2017.23.1.63 PMC5432469

[13] Schiavi MC, Zullo MA, Faiano P, D’Oria O, Prata G, Colagiovanni V, et al. Retrospective analysis in 46 women with vulvovaginal atrophy treated with ospemifene for 12 weeks: improvement in overactive bladder symptoms. Gynecol Endocrinol. 2017;33:942–5. 28490209 10.1080/09513590.2017.1323859

[14] Dezzutti CS, Brown ER, Moncla B, Russo J, Cost M, Wang L, et al. Is wetter better? An evaluation of over-the-counter personal lubricants for safety and anti-HIV-1 activity. PLoS ONE. 2012;7:e48328. 23144863 10.1371/journal.pone.0048328 PMC3492332

[15] Takacs P, Kozma B, Erdodi B, Jakab A, Larson K, Poka R. Zinc-containing vaginal moisturiser gel improves postmenopausal vulvovaginal symptoms: a pilot study. J Menopausal Med. 2019;25:63–8. 31080791 10.6118/jmm.2019.25.1.63 PMC6487289

[16] Honoré LH, Salkie ML, Jajczay FL. The influence of anatomical site and hormonal status on the copper and zinc levels of human uterine smooth muscle. Clin Biochem. 1986;19:46–8. 3955806 10.1016/s0009-9120(86)80071-6

[17] Houston DMJ, Robins B, Bugert JJ, Denyer SP, Heard CM. In vitro permeation and biological activity of Punicalagin and zinc (II) across skin and mucous membranes prone to herpes simplex virus infection. Eur J Pharm Sci. 2017;96:99–106. 27516148 10.1016/j.ejps.2016.08.013

[18] Hatem S, Ahmed E, Soad M. Contraceptive vaginal suppository containing nonoxynol-9 and zinc acetate salt in a clinical trial. J Drug Delivery Ther. 2013;3(1), 20-24.

[19] Damjanovich P, Sipos AG, Larson K, Cunningham TD, Takacs P, Kozma B. Cervicovaginal lavage fluid zinc level as a marker of vaginal atrophy. Menopause. 2020;27(7):776–9. 32301893 10.1097/GME.0000000000001536

